# Assessment and Accessibility of Phenotypic and Genotypic Diversity of Carrot (*Daucus carota* L. var. sativus) Cultivars Commercially Available in the United States

**DOI:** 10.1371/journal.pone.0167865

**Published:** 2016-12-13

**Authors:** Claire H. Luby, Julie C. Dawson, Irwin L. Goldman

**Affiliations:** Department of Horticulture, University of Wisconsin- Madison, Madison, Wisconsin, United States of America; United States Department of Agriculture, UNITED STATES

## Abstract

Increased use of intellectual property rights over plant germplasm has led to a complicated landscape for exchange among plant breeders. Our goal was to examine phenotypic and genotypic diversity present in commercially available carrot (*Daucus carota* L. var. sativus) germplasm in relation to the freedom to operate—the ability for plant breeders to access and use crop genetic diversity. A collection of 140 commercially available carrot cultivars were grown in replicated field trials in the Madison, WI area in 2013 and 2014. Phenotypic measurements were recorded for leaf and root characteristics. Illumina sequencing was used to conduct genotyping by sequencing analysis on all cultivars to understand the range of genetic diversity present. Additionally, the intellectual property rights associated with each cultivar was noted to determine the freedom to operate. We found that although one-third of the commercially available US carrot cultivars in our study are restricted through some form of intellectual property rights, the genetic and phenotypic variability of the protected cultivars does not represent a completely separate group from the available material. Phenotypic analyses including ANOVA and principal components analysis, suggest that many of the traits differed significantly based on market class, but not by whether the cultivar had freedom to operate. The principal components and F_st_ analyses on the genotyping by sequencing data revealed that carrot market classes (F_st_ = 0.065) and freedom to operate classes (F_st_ = 0.023) were not genetically distinct, and that principle components 1 and 2 account for only 10.1% of the total genotypic variation, implying that cultivated carrot germplasm in the US forms an unstructured population. Our findings suggest that the genetic diversity present in carrot cultivars that have freedom to operate is potentially large enough to support carrot breeding efforts in most market classes given present levels of intellectual property protection.

## Introduction

The genetic diversity within crop species is what humans depend on to ensure food security and the resiliency of our agricultural system to climate change. Access to a wide pool of germplasm has facilitated the development of new cultivars and helped maintain genetic diversity within agriculture systems. However, the 20^th^ century has seen a dramatic transition in the distribution of crop germplasm development and release from the public domain into proprietary structures [1]. Intellectual property rights (IPR) were developed to protect the rights of an inventor while simultaneously fostering innovation. Additionally, IPR can be used to incentivize research and development [2]. However, for crop plant germplasm, proprietary restrictions by fewer entities consolidates control and access to genetic diversity. Subsequently, this threatens the exchange of crop genetic resources necessary for innovation in plant breeding.

Plant breeding, in its most fundamental form, relies on human directed selection in genetically variable populations of plants. Genetic diversity within the population under selection is essential in order to be able to utilize the power of selection. Thus, the ability for plant breeders to access plant genetic diversity—freedom to operate (FTO)–is crucial. However, little is known about how this shift has affected the ability of plant breeders to utilize germplasm and what impact IPR restrictions may have on diversity. We examined what FTO looks like in relation to diversity in a single crop: carrot (*Daucus carota* var. sativus). Carrot is a biennial, outcrossing diploid in the family Apiaceae and is an important vegetable crop, both economically and nutritionally. Additionally, good genomic resources exist, and there are both public and private breeding programs in the U.S. developing F1 hybrid and open pollinated cultivars for commercial sale. Beginning with a set of 140 carrot cultivars commercially available in the United States, we examined the diversity among cultivars offered by seed companies in the United States to explore how different companies are protecting their material with IPR and how protection impacts plant breeders’ access to elite carrot germplasm. Three datasets were utilized: (1) phenotypic diversity on root and shoot characteristics of each cultivar; (2) Illumina genotyping by sequencing (GBS) data for each cultivar; and (3) an accounting of any form of legal protection or IPR associated with each cultivar [3]. In this paper, we describe what proportion of phenotypic and genotypic diversity in commercially available carrot cultivars is legally protected and what is freely available to use in future breeding.

### Intellectual Property Rights and Diversity

There are many ways that plant germplasm can be protected through IPR and each has a slightly different effect on how germplasm can be used. These include utility patents, plant patents, plant variety protection, plant breeders’ rights, contract law, trademarks and trade secrets [4]. The invention of hybrid corn in the 1930s and the application of biotechnology to crop plants in the 1970s led to utilization and legislation of intellectual property rights for crop germplasm [5] and the use of IPR to protect crop germplasm has continued to increase [6]. Pardey et al. [6] analyzed plant variety rights granted in the United States between 1930 and 2008 –including plant patents, plant variety protection certificates and utility patents—and found that 42% of the total number were obtained between 2000 and 2008. Most plant variety protections are now from the private sector [6], highlighting the shift from plant breeding as a predominantly public sector activity toward an increase in private sector activity [7]. Additionally, the global seed industry has become increasingly consolidated so that only a few players hold the majority of these proprietary rights [8]. This trend has implications for how plant germplasm is controlled, distributed and ultimately used in plant breeding.

Agricultural diversity can be measured at many scales, from landscape level diversity to intra species crop diversity. Each measure provides a slightly different characterization of agricultural and crop diversity. To our knowledge, this is the first study to examine intra species diversity from the freedom to operate perspective, using both phenotypic and genotypic analyses. While phenotypic studies have been used by scientists for centuries, the ability to sequence genetic information is much more recent. GBS has become a rapid and cost effective approach for reduced-representation sequencing for use in understanding plant genetics. It utilizes genome-wide molecular marker discovery and genotyping of multiplexed samples to further understanding of heritable genetic factors [9]. We utilized both phenotypic and GBS analysis to understand the genetic relationships and phenotypic diversity of commercially available cultivars of carrot.

### Why Carrot?

Carrot is an important vegetable crop, with an annual crop value of $758M and 35,000 hectares of carrots produced annually in the U.S. [10]. Carrot is important nutritionally as well, providing a majority of both ß-Carotene and a-carotene in the US diet [11]. There are several distinct market classes of carrot based on processing and consumer use; and both private and public breeding programs exist that release cultivars using a variety of different IPR protections.

The center of diversity for wild carrot is in present day Afghanistan, although it is indigenous to Europe, North Africa, and western Asia and is ubiquitous worldwide [12]. The first evidence of carrot used as a food crop is in the Iranian Plateau and the Persian Empire in the 10^th^ century AD [13]. These original carrot roots were purple and yellow in color. From Persia, cultivated carrot spread to surrounding areas. Orange carrots appear to have become popular in the 17^th^ century when Dutch and Spanish paintings began depicting orange carrots in market scenes [14], although orange carrots likely originated much earlier [12]. Banga [15] first hypothesized that orange carrots were initially selected from yellow cultivars and this is supported by genetic analyses [16].

Domesticated and wild carrots are genetically distinct [16, 17]. Domesticated carrots can be divided into two groups [16]: The Eastern/Asiatic (var. *altorubens*) and Western (var. *sativus*) groups, which are genetically distinct [16, 18, 19]. There are also phenotypic differences. The Asiatic types have anthocyanin-pigmented roots and are generally purple, red/pink or orange/yellow in color. Plants are often prone to early flowering and bolt easily. The center of diversity for this group is the Himalayan-Hindu Kush region [12]. The Western sub group evolved slightly later and are characterized by carotenoid-pigmented roots that are orange, yellow or occasionally red or white in color. Roots require extended exposure to cold temperatures in order to produce flowers and are thus more adapted to cooler climates [12]. The center of diversity for this group is Central Asia and temperate European regions [16]. The majority of modern cultivars belong to the Western sub group [12], as do the cultivars included in this study.

As an outcrossing species with cytoplasmic male-sterility, it is possible to develop both open-pollinated and F1 hybrid cultivars. Over the past 50 years, the majority of breeding has focused on the development of F1 hybrid cultivars. Breeders have generally selected inbred lines from existing open pollinated cultivars [20]. Like many outcrossing crops, F1 hybrid cultivars of carrot exhibit heterosis and greater uniformity. F1 hybrids also allow the originator to more easily maintain control of the parent inbred lines by not disclosing the parents used to make a hybrid. However, there are still many open pollinated carrot cultivars sold by seed companies and in use by gardeners and farmers.

Carrot can be classified into several market classes based on shape and use. These include: Imperator/Cut-and-Peel (longest type), Nantes, Danvers, Chantenay, Parisienne (shortest type), Amsterdam, Kuroda, Flakee, Belgian and Berlicum [20]. While the majority of cultivars are orange-rooted in color, there are also purple, yellow, red and white-rooted carrots that accumulate a variety of secondary compounds, notably carotenoids and anthocyanins. However, despite phenotypic differences among market class and root color, several studies have suggested that Western/European carrot germplasm forms an unstructured population, meaning that there is not significant genetic separation between groups [16, 18, 19, 21]. Additionally, orange carrots form a sister clade with all other cultivated carrots, suggesting that orange was selected from other colors of cultivated carrots [16].

Carrot breeding programs exist in both the public and private sectors and cultivars are released in different ways. Historically, F1 hybrids (which were protected through maintaining the inbred lines as a trade secret) and material transfer agreements were the only mechanisms of protection used in carrot and the majority of material had FTO for plant breeding. The use of other forms of IPR is relatively recent in carrot, including utility patents, contracts, and ‘bag-tag’ licenses [3]. The use of more and different types of IPR provides a unique point in history to examine the effect of IPR on FTO in this crop, since there are some cultivars that are protected and some that are still freely available.

## Materials and Methods

Through consultation with carrot breeders in the public and private sectors as well as through seed catalogs, we identified 175 carrot cultivars for sale in 2013. We were able to obtain untreated seed of 140 F1 hybrid and open-pollinated carrot cultivars that were commercially available in the United States in 2013 (Table 1, also cited in [3]). Seed of each cultivar was sown in replicated plots on certified organic land at Tipi Organic Produce in Evansville, WI (42.78°N, 89.30°W) and Elderberry Hill Farm in Waunakee, WI (43.18°N, 89.38°W) in the summers of 2013 and 2014. We worked closely with the owners of these farms on this project. Steve Pincus and Beth Kazmar own Tipi Organic Produce and gave permission to collaborate with us on this study. Eric Elderbrock leases land for Elderberry Hill Farm from John Binkley. Both gave permission for us to conduct research at this location. The field study did not involve endangered or protected species.

**Table 1:** 140 of the 175 carrot cultivars commercially available in 2013 (also described in [3]). Table includes the company where seed was obtained from, the color (o = orange, p = purple, r = red, y = yellow, w = white), type (H = F1 hybrid, OP = open pollinated), freedom to operate for plant breeding (n = no FTO, y = has FTO), the market class, and the tip shape (r = majority of tips rounded, t = majority of tips tapered, m = cultivar had a mix of rounded and tapered roots).

Cultivar	Company	Color	Type	FTO	Market Class	Tips
Abledo	Seminis	o	H	n	Chantenay	m
Achieve	Seminis	o	H	n	Flakee	m
Adelaide baby	Kitchen Garden Seeds	o	H	y	Amsterdam	r
Amarillo^b^	Baker Creek	y	OP	y	Danvers	t
Amsterdam 2	Fedco	o	OP	y	Amsterdam	r
Apache	Nunhems	o	H	n	Imperator	t
Arrowhead	Sakata	o	H	n	Imperator	t
Atlas	Johnny’s Selected Seeds	o	OP	y	Parisienne	r
Atomic red	High Mowing Organic Seeds	r	OP	y	Danvers	t
Autumn King	Annie’s Heirloom Seeds	o	OP	y	Nantes	m
Baby Babette	Renee’s Garden Seeds	o	H	y	Nantes	r
Baltimore	Vermont Bean Seed Company	o	H	y	Belgian	m
Bambino	Sustainable Seed	o	OP	y	Amsterdam	m
Bastia	Bejo	o	H	n	Belgian	t
Berlicum 2	Baker Creek	o	OP	y	Berlicum	t
Big Sur	Nunhems	o	H	n	Danvers	t
Big Top	Burpee	o	H	y	Chantenay	m
Bilbo	Veseys	o	H	y	Nantes	r
Bolero	Johnny’s Selected Seeds	o	H	y	Nantes	m
Brilliance	Reimer Seeds	o	OP	y	Nantes	r
Burpee A1	Burpee	o	H	y	Imperator	m
Candysnax	Nunhems	o	H	n	Imperator	t
Caracas	Johnny’s Selected Seeds	o	H	y	Chantenay	m
Carson	Bejo	o	H	n	Chantenay	m
Cellobunch	Seminis	o	H	n	Imperator	t
Chantenay Royal	Reimer Seeds	o	OP	y	Chantenay	m
Choctaw	Nunhems	o	H	n	Imperator	t
Coral II	Evergreen Seeds	o	H	y	Chantenay	r
Cosmic Purple	High Mowing Organic Seeds	p	OP	y	Danvers	t
Creampak	Nunhems	w	H	n	Imperator	t
Crème de Lite	Nunhems	w	H	n	Danvers	t
Crispy Cut	Nunhems	o	H	n	Imperator	t
Cumbre	Nunhems	o	H	n	Chantenay	r
Cupar	Bejo	o	H	n	Chantenay	m
Damco	Osbourne Seed	o	H	y	Amsterdam	r
Danvers 126 Half-long	Burpee	o	OP	y	Danvers	t
Danvers 126	High Mowing Organic Seeds	o	OP	y	Danvers	t
Deep Purple	Johnny’s Selected Seeds	p	H	y	Danvers	t
Dominion	Seminis	o	H	n	Belgian	t
Dragon	High Mowing Organic Seeds	p	OP	y	Danvers	t
Early Milan Nantes	Turtle Tree	o	OP	y	Nantes	m
Envy	Seminis	o	H	n	Danvers	m
Flakkee	Reimer Seeds	o	OP	y	Flakee	m
Flyaway	Osbourne Seed	o	H	y	Nantes	r
Hilmar	Osbourne Seed	o	OP	y	Danvers	m
HoneySnax	Nunhems	o	H	n	Imperator	t
Imperator58	Reimer Seeds	o	OP	y	Imperator	t
Ingot	Sakata	o	H	n	Nantes	t
Interceptor	High Mowing Organic Seeds	o	H	y	Imperator	t
Inverness	Kitchen Garden Seeds	o	H	y	Imperator	t
Invicta	Osbourne Seed	o	H	y	Nantes	r
James Scarlet	Reimer Seeds	o	OP	y	Danvers	t
Jaune du Doubs	Fedco	o	OP	y	Danvers	t
Jeannette	High Mowing Organic Seeds	o	H	y	Nantes	r
Jerada	Osbourne Seed	o	H	y	Nantes	r
Juwarot	Bountiful Gardens	o	OP	y	Danvers	m
King Midas	Renee’s Garden Seeds	o	OP	y	Danvers	r
Kuroda Long	Reimer Seeds	o	OP	y	Kuroda	m
Kuroda Nova	West Coast Seeds	o	OP	y	Kuroda	m
Laguna	Nunhems	o	H	n	Nantes	r
Legend	Seminis	o	H	n	Danvers	t
Little Finger	Baker Creek	o	OP	y	Amsterdam	r
Lunar White^b^	Baker Creek	w	OP	y	Belgian	t
Maverick	Nunhems	o	H	n	Imperator	t
Mellow Yellow	Bejo	y	H	n	Danvers	m
Merida	Osbourne Seed	o	H	y	Nantes	r
Mignon	West Coast Seeds	o	OP	y	Amsterdam	r
Mini Sweet	Bountiful Gardens	o	OP	y	Amsterdam	r
Minicor	Turtle Tree	o	OP	y	Nantes	r
Mokum	Bejo	o	H	n	Nantes	m
Muscade^b^	Baker Creek	o	OP	y	Danvers	r
Nantes Half-long	Burpee	o	OP	y	Nantes	m
Nantes Mini-core	Reimer Seeds	o	OP	y	Nantes	m
Nantindo	Osbourne Seed	o	H	y	Nantes	m
Napa	Bejo	o	H	n	Nantes	m
Napoli	Bejo	o	H	n	Nantes	r
Nash’s Nantes	Nash Huber	o	OP	y	Nantes	m
Necoras	High Mowing Organic Seeds	o	H	n	Nantes	r
Nectar	Johnny’s Selected Seeds	o	H	n	Nantes	r
Negovia	High Mowing Organic Seeds	o	H	n	Nantes	r
Nutrired	Osbourne Seed	r	OP	y	Imperator	t
Olympus	Sakata	o	H	n	Imperator	m
Paris Market	Annies Heirloom Seeds	o	OP	y	Parisienne	r
Parisienne^b^	Baker Creek	o	OP	y	Parisienne	r
Parmex^b^	Kitchen Garden Seeds	o	OP	y	Parisienne	r
Pot o Gold	Vermont Bean Seed Company	o	H	y	Nantes	m
PrimeCut	Nunhems	o	H	n	Imperator	t
Prodigy	Pinetree Seeds	o	OP	y	Danvers	m
PS07101441	Seminis	o	H	n	Imperator	t
Purple Haze^b^	Johnny’s Selected Seeds	p	H	y	Danvers	m
Purple Sun	Kitchen Garden Seeds	p	H	y	Danvers	t
Purplesnax^b^	Osbourne Seed	p	H	y	Imperator	t
Rainbow	Bejo	s	H	n	Danvers	t
Red-Cored Chantenay	High Mowing Organic Seeds	o	OP	y	Chantenay	m
Red Samurai^b^	Kitchen Garden Seeds	r	OP	y	Danvers	t
Resistafly	Thompson Morgan	o	H	y	Nantes	m
Rodelika	Turtle Tree	o	OP	y	Danvers	t
Rolanka	Turtle Tree	o	OP	y	Danvers	t
Romance	Nunhems	o	H	n	Nantes	r
Rothild	Cooks Garden	o	OP	y	Danvers	t
Rotild	Renee’s Garden Seeds	o	OP	y	Danvers	m
Round Romeo	Renee’s Garden Seeds	o	OP	y	Parisienne	r
Scarlet Keeper	Fedco	o	OP	y	Danvers	m
Scarlet Nantes	High Mowing Organic Seeds	o	OP	y	Nantes	m
Sherbert	Nunhems	y	H	n	Imperator	t
Shin Kuroda 5^b^	Fedco	o	OP	y	Kuroda	m
Short N Sweet	Burpee	o	OP	y	Chantenay	r
SlimCut	Nunhems	o	H	n	Imperator	t
Snow White^b^	Baker Creek	w	OP	y	Danvers	t
Solar Yellow	Sustainable Seed	y	OP	y	Danvers	t
St. Valery	Baker Creek	o	OP	y	Danvers	t
Starica	Renee’s Garden Seeds	o	OP	y	Nantes	m
Sugarsnax	Nunhems	o	H	n	Imperator	t
SUN255	Nunhems	o	H	n	Imperator	t
Sunrise Red	Evergreen Seeds	r	H	y	Imperator	t
Sweet Treat	Burpee	o	H	y	Danvers	r
Sweetness III	Vermont Bean Seed Company	o	H	y	Nantes	m
Tastypeel	Seminis	o	H	n	Imperator	t
Tendersnax	Nunhems	o	H	n	Nantes	m
Tendersweet	Annie’s Heirloom Seeds	o	OP	y	Nantes	t
Thumbelina^b^	Kitchen Garden Seeds	o	OP	y	Parisienne	r
Tokita’s Scarlet	Evergreen Seeds	o	OP	y	Kuroda	m
Tonda Di Parigi	Baker Creek	o	OP	y	Parisienne	r
TopCut	Nunhems	o	H	n	Imperator	t
Touchon	Cooks Garden	o	OP	y	Nantes	r
Triple Play	Nunhems	o	H	n	Imperator	m
Triton	Osbourne Seed	o	H	n	Imperator	t
UpperCut	Nunhems	o	H	n	Imperator	t
Vitana	Nunhems	o	H	n	Nantes	r
White Belgian^b^	Baker Creek	w	OP	y	Belgian	t
White Kuttiger	Seedrack	w	OP	y	Danvers	t
White Satin	High Mowing Organic Seeds	w	H	y	Belgian	t
Sovereign	UW-Madison	o	OP	y	Chantenay	t
Oranje	UW-Madison	o	OP	y	Danvers	t
Yaya	High Mowing Organic Seeds	o	H	y	Nantes	r
Yellow Sun	Johnny’s Selected Seeds	y	H	y	Chantenay	m
Yellow Bunch	Nunhems	y	H	n	Imperator	t
Yellowpak	Nunhems	y	H	n	Imperator	t
Yellowstone	High Mowing Organic Seeds	y	OP	y	Danvers	t

If any replications of the cultivar exhibited bolting, this is denoted with a ‘^b^’ next to the cultivar name.

### Field Methods

At Tipi Organic Produce in Evansville, Wisconsin, USA, carrots were planted in 3.7 m rows with three rows to each 1 m bed and 1.2 m alleys between ranges. At Elderberry Hill Farm in Waunakee, Wisconsin, USA, carrots were planted in 2.5 m rows with three rows to each 1m bed and 0.6 m alleys between ranges. Carrots were planted using a Planet Junior planter (Planet Junior, Tunkhannock, PA) fitted with a cone seeder attachment. Experimental design was a randomized complete block design with two blocks at Tipi Organic Produce and one block at Elderberry Hill Farm. In 2013, carrots were planted on July 1 and harvested between October 8–11 at Tipi Organic Produce and planted on July 3 and harvested on October 6 at Elderberry Hill Farm. In 2014, carrots were planted on June 26 and harvested between September 23–26 at Tipi Organic Produce and planted on July 10 and harvested between October 7–10 at Elderberry Hill Farm. Carrots were thinned to population densities according to their market class. Spacing was approximately 30 plants per meter for dicer types, 60 plants per meter for fresh market types and novel colors, and 120 plants per meter for Imperator types. In the field, the following characteristics were measured: petiole anthocyanin, top shape/ leaf growth habit, top height, top strength and bolting. The level of petiole anthocyanin accumulation was measured on a scale of 1 to 3 with a ranking of 1 having no anthocyanin, a rank of 2 having some anthocyanin accumulation and a ranking of 3 having nearly completely purple stems. An example of each rank was used for comparison when evaluating. Top shape was given a rank based on the angle of the leaves where they come out of the crown: 1 = 0°–60°, 2 = 61°–120°, 3 = 120°–180° degrees. For top height, 5 plants were measured in cm from crown of root to top of leaf canopy. Bolting tendency was noted as the number of plants per row. Top strength was given a rank of 1–5. 1 was the weakest, tops were easy to break off and very small, 5 was the strongest, tops were robust and root could be pulled out of the ground by the top. An example of each rank was used for comparison when evaluating.

Immediately after harvesting, carrots were packed in paper bags with wood shavings. The paper bag was then placed in a plastic bag with several holes. Roots were stored at 4°C in the dark until the time of sampling, within 5 weeks of harvest. At sampling, the following were measured on 10 carrots from each row: Red/purple on shoulder, green on shoulder, root length, root diameter, root shape, smoothness, tip shape, branching, uniformity, and outer root color. Red/purple on shoulders were measured on a scale of 1–3: 1 = no red/purple, 2 = of the 10 roots, 10–60% of roots had red/purple blush on the shoulder, 3 = of the 10 roots, >60% of roots had red/purple blush shoulders. Green shoulders were also measured on a scale of 1–3: 1 = no green, 2 = of the 10 roots, 10–60% of roots had green blush on the shoulder, 3 = of the 10 roots, >60% of roots had green shoulders. For root length, 10 roots were measured from crown to tip (cm). For root diameter, 10 roots were measured (cm) at the crown- the widest part of the root. Root shape/market class was categorized as: Amsterdam, Berlicum, Chantenay, Danvers, Flakee, Imperator, Kuroda, Nantes, or Parisienne. Smoothness was given a rank of 1–5 based on the smoothness of the exterior (root hairs, large lenticels). An example of each rank was used for comparison. Tips were categorized based on shape: r = majority of roots round, t = majority of roots tapered, m = mix of round and tapered roots. Branching was rated on a 1–3 scale: 1 = no branching of any roots, 2 = of the 10 roots, 10–60% of roots exhibited some branching, 3 = >60% of roots exhibited branching. An example of each rank was used for comparison. Uniformity of type and size was given a 1–5 rating: 1 = very variable, 5 = very uniform. An example of each rank was used for comparison. Root color was classified as orange, purple, red, white or yellow. Samples approximately 1cm thick were taken from the bottom third of the root for soluble solids analysis and samples were stored at -80°C until analysis. Soluble solids samples were thawed and the 10 roots were combined and juiced. Juice was then analyzed on a Fisher Scientific bench-top refractometer (LR45227) in °Brix.

### Genotyping Methods

Four replicates of each of the 145 cultivars were planted in flats in the greenhouse. Five cultivars were included in the genotypic study that were not included in the phenotypic study since treated seed was the only seed available of these cultivars and we could not plant it due to organic certification requirements on the trial farms. After four weeks, leaf tissue from a single plant of each cultivar was harvested into plates for DNA extraction. 40-50mg lyophilized leaf tissue was submitted to the University of Wisconsin-Madison Biotechnology Center. DNA was extracted using the CTAB method as previously described in Saghai-Maroof et al. [22] with minimal modification. Following elution, a final DNA cleaning step was performed using a 1.5:1 by volume ratio of Axygen Clean-Seq beads (Corning Life Sciences, Corning, NY, USA) to extracted DNA sample to remove any remaining inhibitory compounds in the sample. DNA was quantified using Quant-IT PicoGreen fluorescent dye (Thermo Fisher, Waltham, MA, USA).

Libraries were prepared as described in Elshire et al. [23] with minimal modification. 50ng of DNA were quantified by the Quant-IT PicoGreen dsDNA dye (ThermoFisher) and were digested using ApeKI restriction enzyme. Barcoded adapters were then ligated to each sample. Samples were pooled and amplified. Residual adapter dimers were removed from the final library using a 1:1 dilution of Axygen Clean-Seq beads (Corning Life Sciences). Quality and quantity of finished libraries were assessed using an Agilent DNA1000 series chip assay (Agilent Technologies, Santa Clara, CA) and Invitrogen Qubit HS Kit (Invitrogen, Carlsbad, California, USA), respectively. Each library was standardized to 2μM. Cluster generation is performed using a TruSeq Single Read Cluster Kit (v3) and the Illumina cBot, with libraries multiplexed for 1x100bp sequencing using the TruSeq 100bp SBS kit (v3) on an Illumina HiSeq2000. Images were analyzed using CASAVA 1.8.2.

### FTO for Plant Breeding

In order to determine FTO for plant breeding, we collected data on the type of IPR protecting each cultivar. For the purposes of this study, FTO was either present or absent, though it is possible to have gradations of freedom to use a particular seed source. For example, the ability to use germplasm for plant breeding is often accompanied by a contract outlining royalty or intellectual property arrangements for any commercial derivatives. However, for many plant breeders, any restriction on FTO means limited or “no use” of particular germplasm for plant breeding. Thus, we interpreted FTO as either present or absent for this study.

When determining FTO, we noted if any IPR was associated with each cultivar. We considered those that had explicit restrictions on breeding to have no freedom to operate. However, there were many cultivars where it was unclear exactly whether there was a use-restriction on breeding. These included cultivars with some form of ‘bag tag’ license and cultivars that were obtained from a breeder where it was unknown whether the parent company would agree to allow a cultivar to be used in breeding without some type of exclusive contract. Additionally, there were several cultivars with use-restrictions when they were obtained from one company, but that did not appear to have the same use-restrictions when seed of the same cultivars were obtained from a different company. We considered all of these cases to be restrictions on FTO for plant breeding. If a cultivar had no restrictions on use, we considered it to have FTO for plant breeding.

### Data Analysis

#### ANOVA

A mixed model ANOVA was conducted in R using the lme4 [24] and lmerTest [25] packages on the phenotypic trait data with cultivar, location, year and interaction effects considered as random and freedom to operate and market class as fixed effects in the model. The significance of random effects was determined using a likelihood-ratio test (LRT), with an assumed *χ*^2^ distribution for the LRT statistic under the null hypothesis that the variance of the random effect was zero [26]. The significance of fixed effects was determined using an F statistic. We included several variables that were measured as a rank in the ANOVA analysis. By analyzing the ranking data as a numerical variable, we incorporated the ability to build the experimental design into the model structure.

LS means for each phenotypic trait were calculated in R using the lsmeans package [27] and were calculated from the following model with cultivar as the only fixed effect and location, year and the interactions as random effects.

#### Heritability

Broad sense heritability, the extent to which phenotype is determined by genotype, was estimated using the lme4 package in R. Variance components for genotype by location by year, genotype by year, genotype by location and genotype were estimated for each trait. Using the equations [28]:

$$Golbal\;H^{2}=V_{G}/(V_{G}+\;V_{GL}/loc\;+V_{GY}/\#yr+V_{GLY}/\left(\#yr\times \#loc\right)+V_{e}/\left(\#rep\times \#yr\times \#loc\right))$$

$$Location\;H^{2}=V_{G}/(V_{G}+V_{GY}/\#yr\;+V_{e}/\left(\#rep\times \#yr\right))$$

$$Site\;H^{2}=\;V_{G}/\left(V_{G}\;+\;Ve/2\right)$$

#### Phenotype Principal Components Analysis (PCA)

We used the FactoMineR package [29] in R to conduct a PCA on the scaled and centered variety by trait matrix of LS Means for each of the phenotypic traits measured. Traits included: soluble solids, root length, root diameter, top height, red/purple shoulders, green shoulders, branching, smoothness, uniformity, and petiole anthocyanins.

#### Genotype PCA

Raw data from the Illumina GBS run was analyzed by first using the SNP calling pipeline, "DiscoverySNPCallerPlugin", in Tassel Version: 4.3.13 [30]. SNPs were called using an earlier draft of the carrot genome later released under GenBank accession LNRQ01000000.1 [16]. Coordinates have been adjusted to reflect the published genome. The raw number of SNPs was 370,835. After filtering, 63,807 SNPs were used for the analysis.

Population structure was evaluated with PCA of the centered and scaled variety by SNP marker matrix (SNPs coded as 0 or 2 = homozygous for reference or alternate allele, 1 = heterozygous) using the FactoMineR package in R [29]. Confidence ellipses were drawn for the 95% confidence level using the function coord.ellipse in FactoMineR. The use of principle components has been found to be an appropriate method for characterizing population structure in collections with molecular marker data and relies on fewer assumptions about population history than methods such as STRUCTURE [31, 32]. Individuals missing more than 50% of data were eliminated from the analysis. Missing data was imputed with the population mean. We imputed with both the population mean and mode and found that there was no difference. Wright’s F-statistics and observed heterozygosity were computed as a measure of population differentiation between all pairs of market classes and between cultivars with FTO and without FTO, using the R package adegenet [33, 34] and hierstat [35].

## Results

### ANOVA Analysis

There were a total of 140 cultivars included in the field trial portion of the study (Table 1). Root phenotype observed in field trials was generally consistent with the market class designated by the company, so we used the company categorization for analyses. Of the 140 cultivars, 95 had FTO, 77 were F1 hybrids, and ten market classes and five color classes were represented.

Phenotypic traits were run as the response variable for the mixed model ANOVA (Table 2). For soluble solids, market class was the only significant effect in the model, suggesting that there were significant differences in soluble solids among market classes. Effects of cultivar, replication and market class were significant for the ANOVAs with root diameter and top shape as the response variables. The ANOVAs with purple shoulder and green shoulder as response variables had significant effects of cultivar and market class, suggesting there were differences among market classes for these traits. The top strength variable had significant effects of cultivar, replication, FTO and market class. All of these responses are expected due to differences among cultivars for most traits measured.

**Table 2:** Results from an Analysis of Variance comparing effects of the model with different phenotypic traits as response variables. Chi square value is reported for random effects and F statistic is reported for fixed effects.

	Soluble solids	Root length	Root diameter	Top height	Uniformity	Smoothness	Branching	Top strength	Top shape	Purple shoulder	Green shoulder
*chi square*											
Cultivar	1.73	16.19***	35***	30.80***	10.80***	11.40***	6.58*	37.98***	18.7***	10.10**	101***
Location	0.75	3.74	0	4.55e-12	2.96E-12	1.00e-11	2.27e-12	2.81	0.09	0	0.11
Year	2.77	0.89	0.01	5.46e-12	9.09E-13	6.56*	2.10	0	0	0.13	0.37
Cultivar: location	1.41e-11	7.78**	2.76	0.12	2.27E-13	0	2.27e-12	2.73	0	0.08	6.82E-13
Cultivar: year	0	0.08	0	0.15	9.37**	1.69	3.94*	0.07	4.55E-12	1.17	6.14E-12
Cultivar: location: year	0	8.39**	0.03	9.20**	0	0.57	3.64e-12	0.96	6.82E-11	7.58E-4	6.82E-12
Rep	3.47	38.83***	210***	115***	26.60***	0.33	0.16	35.44***	9.96**	6.82e-12	8.41E-12
*F value*											
Freedom to Operate	0.03	8.09**	0.09	0.08	8.39**	18.09***	0.40	5.27*	2.67	1.90	0.21
Market Class	3.71***	41.89***	20.98***	9.33***	1.87	5.54***	5.98***	12.20***	2.17*	2.10*	4.15***

P value significance is denoted at the following levels ‘***’ <0.001, ‘**’ <0.01, ‘*’ <0.05.

The model with uniformity as the response variable exhibited significant effects of cultivar, replication, FTO and the interaction of cultivar x year. The cultivar x year interaction suggests that there was a change in rank or magnitude of cultivar for uniformity between years. Similar to the results of the uniformity model, branching also exhibited significant effects of cultivar, the interaction of cultivar x year and market class. The model with smoothness as the response variable exhibited significant effects of cultivar, year, FTO and market class. The significance of year suggests that the average smoothness ranking for each year was significantly different but there was not a significant interaction with any other effect. The significance of the FTO and market class variables suggests that there were differences in smoothness of roots among market classes and those with and without FTO.

With top height as the response variable, cultivar, the interaction of cultivar x location x year, replication and market class were all significant effects. The significance of the cultivar x location x year interaction was likely due to magnitude differences among means, primarily due to the Elderberry Hill location in 2014. For the ANOVA with root length as the response variable, cultivar, the interactions of cultivar x location and cultivar x location x year, replication, FTO and market class were all significant. We conducted a Spearman’s rank correlation test on the rank of the root length measurement for each cultivar for each location and found that there was a significant correlation among ranks between locations (rho = 0.67, p < 2.2e-16). This suggests that while there was some change in rank of cultivars between locations, ranks in the two locations were still significantly correlated. The greatest changes in rank were for cultivars with root lengths in the middle with fairly good correlation between locations for cultivars with the shortest and longest root lengths. Based on this result we did not separate the two locations for the principle component analysis.

### Heritability

Broad sense heritability estimates from this core collection of commercially available US carrot cultivars provides a general understanding of the degree of genetic control of many market traits. Root length, root diameter, top height, green shoulders, and petiole anthocyanin all had global heritability estimates over 0.80 (Table 3). Soluble solids had the lowest heritability estimates of any trait. It is expected that such broad sense heritability estimates will be much larger than those estimated from narrow sense heritability calculations using the additive genetic variance.

**Table 3:** Broad sense heritability estimates (H^2^) for phenotypic traits measured based on the global variation in phenotype, the location (over both years) and site specific variance.

Trait	Global H^2^	Location H^2^	Site H^2^
Soluble solids	0.43	0.27	0.16
Root length	0.91	0.92	0.86
Root diameter	0.90	0.87	0.77
Top height	0.82	0.77	0.65
Smoothness	0.73	0.61	0.50
Branching	0.63	0.50	0.39
Purple shoulders	0.59	0.48	0.36
Green shoulders	0.92	0.85	0.75
Petiole anthocyanin	0.89	0.90	0.82

### Phenotypic PCA

**Fig 1:**
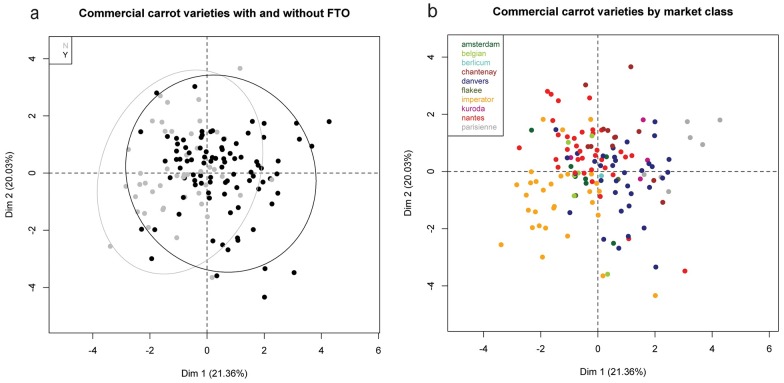
Principal Components Analysis of phenotypic data estimated using LS means estimates and showing (a) PC 1 and 2 for commercial carrot cultivars color coded based on freedom to operate (FTO). A grey dot indicates the cultivar has no FTO for plant breeding and a black dot indicates the cultivar has FTO for plant breeding. Confidence ellipses shown are the 95% confidence level for the group with the corresponding color. (b) Shows the same PCA but is color coded based on carrot market class.

**Fig 2:**
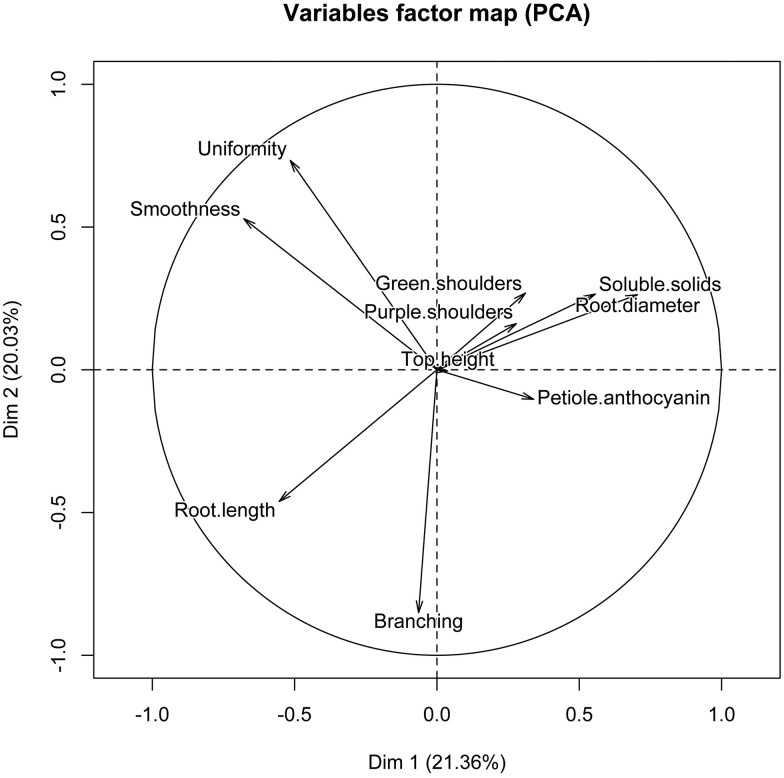
Factor loadings showing contributions of each trait to PC 1 and 2 for the principal components analysis of the phenotypic data.

We conducted a PCA using the LS means estimates for the phenotypic data. Even though there was a significant interaction of cultivar x location for root length, when we ran the PCA analysis separately with the root length estimates for each location, the resulting graphs were very similar and did not change our results or conclusions drawn from the PCA analysis. Thus, we determined that we would use the combined LS means estimates. PC 1 and 2 accounted for 41.39% (Fig 1). Both graphs show some clustering by market class and FTO, however there is significant overlap among market classes and FTO classes. Imperator types, the most protected market class, tend to cluster in the lower left corner of the PCA, while Parisienne types cluster in the upper right corner. Root length, root diameter, and soluble solids strongly influence PC 1 while branching, smoothness and uniformity influence PC 2 (Fig 2).

### GBS Analysis

**Fig 3:**
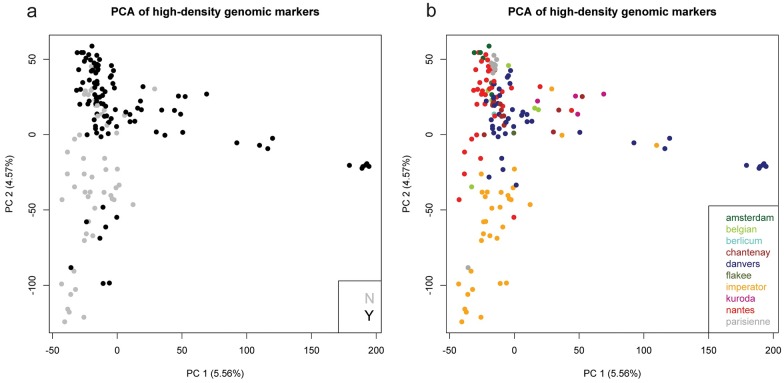
Principal Components Analysis of high density genomic markers showing (a) PC 1 and 2 for commercial carrot cultivars color coded based on freedom to operate (FTO). A grey dot indicates the cultivar has no FTO for plant breeding and a black dot indicates the cultivar has FTO for plant breeding. Confidence ellipses shown are the 95% confidence level for the group with the corresponding color. (b) Shows the same PCA but is color coded based on carrot market class.

The Illumina GBS data were of high quality, with Phred scores above 30 (more than 99.9% base call accuracy), and we were able to use it to conduct a PCA. The average heterozygosity across all samples was 15%. The genotypic PCA indicates that the first two PC only account for about 10.13% of the total variation (Fig 3). It appears that genetic variation related to root color is the major contributor to PC 1; roots to the far right are all purple or red in color while orange or yellow roots are to the left. Similarly, genetic variation for root length likely contributes to PC 2 as the Parisienne types cluster in the upper left and the imperator types are on the other end of the axis, in the bottom left. The amount of genetic variation accounted for by the first two PCs is also relatively low, suggesting an unstructured population. In addition to the small percentage of total variation accounted for by the first two PC, there is significant overlap between clusters for both market class and FTO group. The F_st_ analysis suggests weak differentiation between market classes or FTO groups (Table 4). The average F_st_ across all market classes was 0.065. The strongest differentiation was between Berlicum and Flakee (F_st_ = 0.214), Berlicum and Kuroda (F_st_ = 0.190), and Kuroda and Amsterdam (F_st_ = 0.178) market classes. The average measure of inbreeding within groups (F_is_) was 0.48, which is in the expected range as our dataset includes inbred, hybrid and population varieties. An F_is_ of zero would indicate Hardy-Weinberg equilibrium and an F_is_ of 1 would indicate complete selfing. Within-group observed heterozygosity ranged from 0.111 for Amsterdam to 0.206 for Belgian. The market classes with the largest numbers of cultivars, Danvers, Imperator and Nantes, all had within-group observed heterozygosity between 0.144 and 0.176. These values are greater than the pairwise Fst values, indicating that within population diversity is a more important contributor to overall diversity than population differentiation in this sample. The F_st_ between cultivars with FTO and without FTO was 0.023.

**Table 4:** F_st_ estimates for differentiation based on market class of the genotyping by sequencing data. Darker shading indicates a greater F_st_ estimate.

Market Classes	Danvers	Imperator	Nantes	Chantenay	Parisienne	Belgian	Flakee	Amsterdam	Kuroda	Berlicum
**F**_**is**_	0.558	0.434	0.429	0.498	0.387	0.350	0.3678	0.521	0.380	NA
**Ho**	0.144	0.169	0.176	0.138	0.157	0.206	0.1224	0.111	0.149	0.158
**Imperator**	0.022									
**Nantes**	0.030	0.036								
**Chantenay**	0.023	0.027	0.024							
**Parisienne**	0.038	0.044	0.035	0.049						
**Belgian**	0.024	0.036	0.021	0.060	0.088					
**Flakee**	0.010	0.017	0.015	0.034	0.066	0.090				
**Amsterdam**	0.038	0.061	0.034	0.085	0.108	0.098	0.131			
**Kuroda**	0.020	0.042	0.039	0.075	0.139	0.146	0.120	0.178		
**Berlicum**	0.014	0.021	0.019	0.053	0.079	0.099	0.214	0.115	0.190	

Overall: Fst = 0.0672, Fis: = 0.484, Ho = 0.153, Ht = 0.318

## Discussion

Historically, breeders would use finished cultivars from other breeding programs along with their own breeding material to develop new cultivars. While increasing IPR is the likely result of this practice—as breeders and companies work to prevent appropriation of their material—it is still possible to access a diversity of carrot germplasm. We found that while there are significant restrictions to breeding with commercially available germplasm (about one-third of the germplasm used in this study was restricted in some way), the amount that remains available does encompass many commercially valuable traits. This may be an encouraging finding for future carrot breeding efforts, provided that the number and scope of intellectual property restrictions for carrot cultivars does not increase in the future. Anecdotal data we collected as part of this project suggested, however, that intellectual property protections for carrot cultivars are increasing, at a fairly rapid rate [3]. This is one of the first studies, to our knowledge, that examines the demonstrable impact of IPR on access to crop genetic diversity.

The ANOVA and PCA phenotypic analyses suggest that many of the traits measured differed significantly based on market class, which would be expected given that breeders typically treat market classes as separate breeding targets. Many of the traits measured are used to define market classes (root length and diameter) or have been selected for in breeding programs due to consumer preference (soluble solids, smoothness) or production characteristics (top strength, top shape). However, even though breeders and seed companies treat market classes as separate, there has been significant crossing among market classes within breeding programs and thus there may be little genetic differentiation among the classes. FTO was significant for uniformity, with the group of cultivars without FTO having significantly higher uniformity rankings than those with FTO. Cultivars without FTO were all F1 hybrids whereas cultivars with FTO were a mix of open pollinated and F1 hybrids. Since F1 hybrids tend to be more uniform, this could account for the significance of this effect. Uniformity is likely environmentally influenced, as it exhibited a significant cultivar x year interaction.

Both FTO and market class were highly significant for smoothness, suggesting that there were significant differences among cultivars with and without FTO and in different market classes. The market classes with the highest smoothness rankings were Imperator, Nantes and Chantenay. Nantes and Imperator were also the market classes with the highest number of restricted cultivars. Root length exhibits a similar trend—effects of both FTO and market class were significant for this trait in the ANOVA. As is clearly visualized by the phenotypic PCA (Fig 1), the Imperator market class (longest roots) clusters in the lower left corner and the Parisienne types (shortest roots) cluster in the upper right with intermediate length types in the middle. The upper left also contains the majority of cultivars with no FTO. The top strength model also had significant effects for both FTO and market class. Market classes such as Amsterdam, Nantes, and Parisienne types had lower top strength rankings. Bulkier root types such as Chantenay and Belgian had the highest top strength ranking. The group of cultivars with FTO had higher top strength ranking than the group without FTO. Again, this is likely because the Imperator and Nantes types, the market classes most represented in the no FTO group, generally had weaker tops.

While broad sense heritability does not provide information on the degree to which a trait is controlled by additive gene effects, it does give a sense of what proportion of the variance observed is the result of genetic factors. The heritability calculations suggest that these traits all have some genetic factors contributing to observed phenotypic variation.

Our study found no distinctive genetic population structure, which is consistent with other genetic analyses of Western/European carrot germplasm [16, 17, 19, 21]. This suggests that despite FTO constraints on certain cultivars and market classes, it may be possible to select similar types from unprotected cultivars, even those in a different market class. While this means that IPR may not be significantly restricting access to genetic diversity in carrot at this point, it does call into question the use of IPR to protect specific traits or cultivars that may differ only slightly genetically from one another. This is relevant since the cultivars with and without FTO are socially structured by company/breeder. Cultivars with IPR were distributed by 7 of the 24 companies we obtained seed from. The 4 companies that sold 85% of no-FTO cultivars protected all of the cultivars that they sold. These were also the companies with in-house carrot breeding programs, suggesting that there is a trend toward more protection on new cultivars.

The Imperator market class is the focus of most U.S. based fresh market breeding programs and was developed from a Nantes x Chantenay cross in the 1920s. This is also the most protected market class—of the 30 Imperator cultivars included in this study, only 4 orange cultivars had FTO for breeding, rendering the commercial material in this market class basically unavailable. Interestingly, many Imperator-type inbred lines are publicly available from the USDA-ARS carrot breeding program. Some of these are likely used in the production of proprietary hybrids, although we do not have data on the inbred parent lines used in producing the hybrids included in this study. Thus, someone interested in starting a breeding program with a focus on the Imperator market class could do so using the material available from the USDA, but not generally from the cultivars produced and sold by seed companies that were bred using the same material. Additionally, there appears to be little genetic basis for carrot market class and breeders routinely make crosses between classes. Since the population is genetically unstructured, it may be possible to successfully breed Imperator type carrots from other germplasm, or to use publically available inbred lines and develop a F1 hybrid trialing program. The restrictions placed on Imperator types highlight the trend toward increasing use of IPR on cultivars with the most market value.

This analysis highlights the increase in use of IPR over carrot germplasm, especially for cultivars and market classes that are the focus of breeding programs. The majority of new cultivars are F1 hybrids. While the inbred/F1 hybrid breeding method in itself is a form of protection—by being able to keep inbred lines a trade secret—F1 hybrids also tend to come with additional FTO restrictions. The majority of the F1 hybrid cultivars also had IPR or FTO restrictions associated with them (47/77).

Currently, one of the biggest impacts of IPR on plant diversity appears to be the chilling effect on use of protected material in breeding [3]. The uncertainty surrounding whether one has the ability to use a specific cultivar or line in breeding is its own type of restriction. Additionally, the use of IPR on newer cultivars and market classes that tend to be the focus of breeding programs in the US highlights the trend toward more protection, consolidation and siloing of germplasm within a breeding program and less exchange amongst breeders. The longer term impacts that this will have on inter and intra species diversity of the agricultural landscape remains to be seen [36].

## Conclusions

Our findings suggest that the genetic diversity present in carrot cultivars that have FTO is large enough to support carrot breeding efforts given present levels of intellectual property protection. While market classes demonstrate phenotypic and some genetic differences, they are largely a construct of breeders and can be malleable. There is not significant genetic differentiation between most market classes, which suggests that market classes with mostly restricted material could potentially be created by breeding from other market classes. The plasticity of market class is an area that could receive more attention from researchers. A subset of the commercially available US carrot cultivars is restricted through IPR, but the subset of genetic and phenotypic variability they represent does not represent totally unique variation. Therefore, carrot breeding efforts may make use of FTO variation to get to the same endpoint, at least with the current state of restrictions. As those continue to increase, this ability to utilize variation may change.

Carrot may represent a crop that is average with respect to FTO and non FTO variation at present. There is significant potential to apply this technique and analysis to other crops to determine the freedom to operate. There are certainly crops, generally those with greater market value, with significant restrictions due to use of IPR. Essential to understanding the effect that IPR has on utilization and exchange of diversity will be tracking the trends in breeding of various species of crop plants and examining the landscape level effects on diversity and agricultural systems. Utilization of diverse genetic material will ensure that we have plant cultivars suitable for sustainable and resilient agricultural systems.

## Supporting Information

S1 Table LSMeans estimates for phenotypic traits measured on carrots grown at Elderberry Hill Farm and Tipi Organic Produce in the summers of 2013 and 2014. Traits include Soluble Solids, Root Length, Root Diameter, Top Height, Petiole Anthocyanins, Green Shoulders, Purple Shoulders, Branching, Smoothness, Uniformity, Top Shape, Top Strength.
(XLSX)

S2 Table File of filtered SNP data. Raw data from the Illumina GBS run was analyzed by first using the SNP calling pipeline, "DiscoverySNPCallerPlugin", in Tassel Version: 4.3.13 [30]. SNPs were called using an earlier draft of the carrot genome later released under GenBank accession LNRQ01000000.1 [16]. Coordinates in this file have been adjusted to reflect the published genome. The raw number of SNPs was 370,835. After filtering, 63,807 SNPs were used for the analysis.
(GZ) Click here for additional data file.

## Abbreviations

FTO: Freedom to operate
GBS: Genotyping by sequencing
IPR: Intellectual property rights
PCA: Principal components analysis
